# Serum lipid profiles and risk of colorectal cancer: a prospective cohort study in the UK Biobank

**DOI:** 10.1038/s41416-020-01143-6

**Published:** 2020-11-03

**Authors:** Zhe Fang, Mingming He, Mingyang Song

**Affiliations:** 1 Department of Epidemiology, Harvard T.H. Chan School of Public Health, Boston, MA USA; 2 Department of Medical Oncology, Sun Yat-sen University Cancer Center, State Key Laboratory of Oncology in South China, Collaborative Innovation Center for Cancer Medicine, Guangzhou, Guangdong China; 3 Division of Gastroenterology, Massachusetts General Hospital and Harvard Medical School, Boston, MA USA; 4 Department of Nutrition, Harvard T.H. Chan School of Public Health, Boston, MA USA; 5 Clinical and Translational Epidemiology Unit, Massachusetts General Hospital and Harvard Medical School, Boston, MA USA

**Keywords:** Risk factors, Gastrointestinal cancer

## Abstract

**Background:**

It remains unclear whether serum lipids influence colorectal cancer (CRC) risk.

**Methods:**

We conducted a prospective cohort study of 380,087 adults aged 40–69 years in the UK Biobank. Serum high-density cholesterol, low-density cholesterol, total cholesterol, triglycerides, and apolipoprotein A and B were measured. We used Cox proportional hazard models to estimate the multivariable hazard ratios (HRs) of CRC according to one standard deviation (SD) increment in serum lipids. We conducted subgroup analysis by tumour anatomical subsites.

**Results:**

During a median of 10.3 years of follow-up, we documented 2667 incident CRC cases. None of the lipid biomarkers was associated with the risk of CRC after adjusting for potential confounding factors, including body mass index and waist circumference. When assessed by cancer subsites, serum triglycerides was associated with an increased risk of cancer in the caecum and transverse colon, with the HR of 1.12 (95% CI, 1.00–1.25) and 1.29 (95% CI, 1.09–1.53), respectively; and apolipoprotein A was associated with a lower risk of hepatic flexure cancer (HR, 0.73, 95% CI, 0.56–0.96).

**Conclusions:**

Serum lipid profiles were not associated with colorectal cancer risk after adjusting for obesity indicators. The potential subsite-specific effects of triglycerides and apolipoprotein A require further confirmation.

## Background

Colorectal cancer (CRC) is the third most common malignancy and the second leading cause of cancer death worldwide.^1^ Obesity and poor diet are considered widely to be the major risk factors for CRC.^2^ In obese individuals, adiposopathy, a pathogenic effect due to adipocyte hypertrophy and excessive adipose tissue accumulation, results in abnormal concentrations of circulating lipids through releasing triglycerides (TG), free cholesterol and other body lipids stored in adipocyte and adipose tissues into blood.^3^ Given the close link between obesity and dyslipidaemia, the role of lipids in CRC risk and progression is of interest.^4–6^ Experimental studies showed that serum lipids and lipoproteins may influence carcinogenesis through insulin resistance, inflammation, and oxidative stress pathways.^7,8^ Animal feeding models illustrated that enhanced lipolysis in the visceral depot leads to an increase in free fatty acid (FFA) flux, which stimulates insulin release and reduces insulin sensitivity that may enhance carcinogenesis.^9^ Low-density lipoprotein cholesterol (LDL) enhances intestinal inflammation and CRC progression via activation of ROS and signalling pathways including the MAPK pathway.^10^ A recent study in vitro reported that cholesterol stimulated CRC cell proliferation and inhibited cell apoptosis through the miR-33a-PIM3 pathway.^11^ Nevertheless, epidemiological findings on serum lipids and CRC have been conflicting.^12–14^

A number of studies reported a higher risk of CRC associated with lower levels of high-density lipoprotein cholesterol (HDL), higher levels of LDL and higher levels of TG—markers of dyslipidemia.^15–17^ A recent meta-analysis of 17 prospective studies including 1,987,753 individuals with 10,876 CRC events reported that high levels of TG and total cholesterol (TC) were associated with an increased risk of CRC, whereas HDL might be associated with a decreased risk of CRC.^15^ In contrast, another meta-analysis including 33 case–control, nested case–control and cohort studies reported that high levels of TC, TG and LDL were associated with colorectal adenoma but not with CRC, and no association was observed between levels of HDL and colorectal neoplasia.^18^ One of the potential explanations for the discrepancy may be the different degree of confounding control for obesity indicators, especially visceral obesity that has been suggested as the main driving factor of the association between obesity and CRC risk.^19^

Substantial evidence indicates the etiologic heterogeneity of CRC according to anatomic subsites.^20–23^ The associations of body mass index (BMI) and waist circumference were observed to be more strongly associated with distal than proximal colon cancer.^21,24^ A large health insurance study in Korea indicated the effects of serum TC might vary by CRC subsites.^25^ A case−cohort study reported that the highest tertiles of TC and LDL were significantly associated with increased risks of colon cancer, distal colon cancer, and rectal cancer, but not proximal colon cancer.^26^ However, few studies have examined the role of lipids and lipoproteins in CRC according to more detailed tumour subsites. The majority of previous studies only considered the simplified subclassification of subsites according to proximal colon, distal colon and rectum, without accounting for potential differences within these broad sites.^21^ Also, the findings have been inconsistent, possibly due to the limited power for the analysis of each specific subsite.^27,28^ In addition, it is noteworthy that the incidence and mortality of CRC have been increasing in adults younger than 50 years, the commonly recommended age for starting screening. Such increase in the so-called early-onset CRC has been proposed to be at least partly attributable to the increasing prevalence of obesity.^29,30^ Nevertheless, it remains unknown whether lipids have a particularly strong association with early-onset CRC.

Therefore, leveraging the serum lipid measurements in approximately 400,000 participants in the UK Biobank, we conducted a prospective cohort study to investigate the associations between lipid profiles (HDL, LDL, TC, TG, apolipoprotein A (ApoA) and apolipoprotein B (ApoB)) and risk of CRC, independent of other established and suspected risk factors. We further examined whether the associations differed according to tumour subsites and age of onset, as well as established risk factors of CRC.

## Methods

### Study and participants

**Fig. 1:**
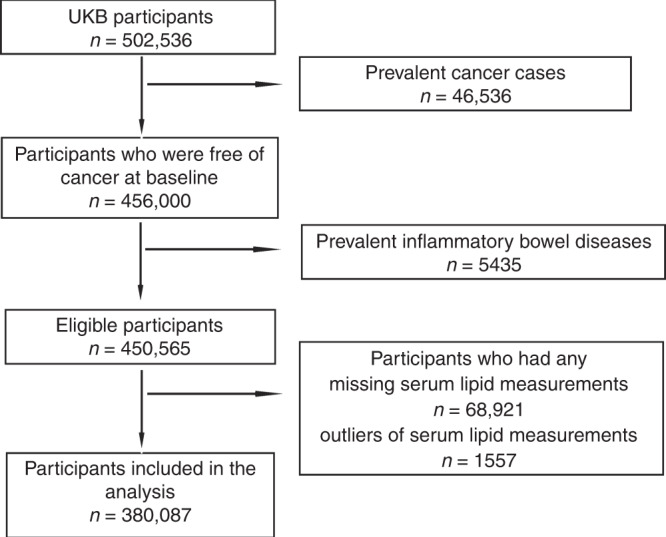
The flow diagram for exclusion and inclusion. We excluded participants who had prevalent cancers, inflammatory bowel diseases including Crohn’s disease according to International Classification of Diseases (ICD-10, 10th revision) codes K50 and ulcerative colitis according to ICD-10 codes K51, any missing serum lipid measurements, and any serum lipid outliers, leaving 380,087 participants in our study.

The UK Biobank is a population-based prospective cohort study of over 500,000 participants aged 40–69 years recruited in 22 assessment centres between 2006 and 2010 throughout the UK.^31^ At baseline visit, participants who signed consent completed self-administered touch screen questionnaire (sociodemographic factors, family history and early life exposures, psychosocial factors, environmental factors, lifestyle and health status), underwent physical measurements and provided a blood sample in the assessment centres (*n* = 502,536).^32^ We excluded participants who had prevalent cancers (*n* = 46,536), inflammatory bowel diseases including Crohn’s disease according to the International Classification of Diseases (ICD-10, 10th revision) codes K50 and ulcerative colitis according to ICD-10 codes K51 (*n* = 5435), any missing serum lipid measurements (*n* = 68,921), and any serum lipid outliers (*n* = 1557) identified using extreme studentised deviate Many-outlier procedure,^33^ leaving 380,087 participants in our study (Fig. 1).

### Assessment of exposure

Serum lipid and lipoprotein levels, including HDL, LDL, TC, TG, ApoA, and ApoB, were available in the UK Biobank and measured by biochemical assays from the blood samples collected at baseline, utilising Beckman Coulter AU5800 Platform. HDL was analysed using enzyme immune-inhibition method; LDL was analysed using enzymatic selective protection method; TC and TG were analysed using enzymatic method; APOA and AOB were analysed using immune-turbidimetric method. Standardised procedures were performed such that each sample was collected, transported, processed and stored in the same way with strict quality assurance and control, aiming to reduce systematic error.^34^ The protocol detailing the handling and storage of biological samples was developed through a wide consultation and peer review in the scientific community, followed by extensive validation.^34,35^ One element of the quality protocol was the bracketing of participant samples with Internal Quality Control (IQC) samples of known high, medium and low concentrations run prior to each batch of participant samples (opening bracket) and after each batch (closing bracket). Only if both the opening and closing IQC results were within the set control limits for the analytical process were participant results validated into the dataset. Third-party IQC materials (from Randox Laboratories and Technopath) were run for each assay to give an unbiased performance assessment of the whole analytical system. The average within-laboratory (total) coefficients of variation (%) across low, medium and high IQC level of HDL, LDL, TC, TG, ApoA, and ApoB were 1.72–1.81, 1.57–1.71, 1.41–1.78, 2.05–2.27, 1.70–2.04, and 2.46–2.68, respectively. Full details of the assay performance have been published.^36^ Moreover, a subsample of 15,457 participants underwent a repeated assessment of lipid biomarkers in 2012–2013 (a median of 4.45 years apart). In the current study, we used the baseline measurements as the main exposure and used the repeated assessments to evaluate reproducibility.

### Assessment of covariates

Sociodemographic (date of birth, gender and race/ethnicity) and lifestyle (smoking, alcohol drinking and physical activity) characteristics were collected using self-completed questionnaires. Each participant was assigned Townsend deprivation index representing the socioeconomic status that corresponds to the postcode of residence. We calculated age from dates of birth and baseline assessment. Physical activity was based on the self-report using the International Physical Activity Questionnaire short form,^37^ and then calculated by summing walking and moderate and vigorous activity, measured as metabolic equivalents (MET min/week). Dietary information (e.g. processed meat intake) was collected via the Oxford WebQ, a web-based 24-h recall questionnaire that was developed specifically for use in large population studies.^38^ Trained nurses measured height, body weight, waist circumference and systolic blood pressure during the initial assessment centre visit. We calculated BMI as weight/height^2^. We collected medical history (physician’s diagnosis of stroke, angina, heart attack, hypertension, diabetes, hypertension), regular aspirin use and ever having CRC screening from the self-completed baseline assessment questionnaire. Health records were used to supplement information recorded at enrolment about previous medical history, family history and medication (http://www.ukbiobank.ac.uk/wp-content/uploads/2011/11/UK-Biobank-Protocol.pdf).

### Assessment of outcome

Date of death was obtained from death certificates held within the National Health Service Information Centre (England and Wales) and the National Health Service Central Register (Scotland). Prevalent and incident cancer cases within the UK Biobank cohort were identified by national cancer registries. The primary outcome was defined as the first diagnosis of incident CRC with ICD-10 codes C18-C20. Proximal colon cancers included those that occurred within the caecum (C18.0), appendix (C18.1), ascending colon (C18.2), hepatic flexure (C18.3), transverse colon (C18.4) and splenic flexure (C18.5). Distal colon cancers included those that occurred within the descending (C18.6) and sigmoid (C18.7) colon. Rectal cancer included those occurred at the rectosigmoid junction (C19) and rectum (C20). Overlapping (C18.8) and unspecified (C18.9) lesions of the colon were also recorded in the cohort.

### Statistical analysis

Baseline characteristics were described among total population, CRC cases and non-CRC cases. Participants contributed person-time from the date of blood draw until the date of the first diagnosis with CRC, loss to follow-up, death or the end of the study (28 February 2019), whichever occurred first. The intraclass correlation coefficients (ICCs, or reliability coefficients) and 95% confidence intervals (CIs) between the baseline measurements and the repeated assessments were computed.^39^

Cox proportional hazard models using attained age as the time scale were used to estimate hazard ratios (HRs) and 95% CIs for the associations of lipid biomarkers with the risk of CRC. Multivariable restricted cubic splines were used to explore non-linear associations and no evidence of deviation from linearity was detected. We assessed the proportional hazards assumption using the Schoenfeld residuals and the interaction test for each of the exposures with the time variable. No violation of the assumptions was found.

In the primary analysis, we treated the exposures as continuous variables, calculated the HRs per 1 standard deviation (SD) increment and reported *P* for trend. We also classified lipids into quintiles and calculated the HRs using the lowest quintile as the reference. We considered three analytic models. The minimally adjusted model only included age at baseline, sex and race (white, non-white). The multivariable-adjusted model further included Townsend deprivation index, height, smoking status (never, former and current), alcohol drinking (never or special occasions only, 1–3 times/month, 1–2 times/week, 3–4 times/week and daily), physical activity (measured by metabolic equivalents), processed meat intake (never, less than once/week, once/week and more than 2 times/week), fasting status, history of cardiovascular diseases (heart attack, angina, stroke and hypertension), history of diabetes, family history of CRC, regular aspirin use (yes/no) and ever having CRC screening (yes/no). The fully adjusted models further included waist circumference and BMI to account for the potential confounding by general and abdominal obesity. For covariates with missing data, we used the missing indicator method for categorical variables and used the mean imputation for continuous variables.

To examine whether the associations vary by tumour subsites (caecum, ascending colon, hepatic flexure, transverse colon, splenic flexure, descending colon, sigmoid colon and rectum) and age of CRC onset (early-onset: <50, middle-onset: 50–59, late-onset: ≥60 years), we used the contrast test method based on the estimates and standard errors of subtype-specific log(HRs) obtained from the Cox proportional hazards model.^40^ In the analysis of early-onset CRC, participants who entered the cohort later than 50 years old were excluded and the outcome event was defined as the incidence of CRC diagnosed before the age of 50 years. Similar exclusions and censoring were done for the analysis of middle- and late-onset CRC.

In addition, we conducted stratified analysis according to age at baseline (<50, 50–59, ≥60 years), sex (male, female), BMI category (<25.0, 25.0–29.9, ≥30.0 kg/m^2^), sex-specific tertiles of waist circumference, smoking status (never, former and current smokers), alcohol drinking (never, 1–3 times/month, 1–2 times/week, 3–4 times/week and daily), quintiles of physical activity, history of cardiovascular diseases (yes/no) and aspirin use (yes/no). Effect modification on the multiplicative scale was tested by including a product term of the stratified factors with serum lipid levels (per 1 SD increment) in the model and *P* for interaction was reported. We performed all the subgroup and stratified analyses using the fully adjusted model and calculated the HRs per 1 SD increment for each of the lipid biomarkers.

To test the influence of reverse causality, we conducted analyses after exclusions of cases diagnosed within 2 and 5 years, respectively, after the blood draw. We also conducted two sensitivity analyses by further excluding participants with any missing values of covariates and additionally adjusting for hormone replacement use (women only) and intake of red meat (beef, lamb and pork), fruits and vegetables.

All analyses were performed using SAS 9.4 (SAS Institute Inc., Cary, NC, USA). Two-sided *P* < 0.05 was considered significant for the primary analysis. To account for multiple testing, a stringent *P* value < 0.005 was used as the cut-off for the secondary analyses (including the subgroup analyses according to subsite and age of diagnosis of CRC and the stratified analyses).^41^

## Results

Among 380,087 participants (179,401 males, 200,686 females) followed for a median of 10.3 years (interquartile range, 9.3–10.7; overall range, 0–12.2), we documented 2667 cases of CRC (1555 in males and 1112 in females). The mean (SD) age of participants at enrolment was 56.2 (8.1) years and 93.9% were white. The baseline characteristics of participants were presented in Table 1. Compared with the total cohort, participants with incident CRC were older, had a higher BMI, higher weight circumference and less intense physical activity. Moreover, they were more likely to be male, white and smokers, to consume processed meat twice or more a week and alcohol daily, and to have family history of CRC and prevalent cardiovascular diseases and diabetes.

**Table 1:** Age-adjusted baseline characteristics of the study population in the UK Biobank (all variables are age-standardized except age itself. Mean (standard deviation) is presented for continuous variables).

Characteristics	Total cohort (*n* = 380,087)	Colorectal cancer cases (*n* = 2667)
Age, years	56.2 (8.1)	60.8 (6.5)
Male (%)	47.2	55.0
Race/ethnicity (%)		
White	93.9	95.1
Non-white	5.6	4.4
Missing	0.5	0.5
Townsend deprivation index	−1.3 (3.1)	−1.3 (3.2)
Height, cm	168.7 (9.3)	169.8 (9.1)
BMI, kg/m^2^	27.5 (4.8)	27.8 (4.6)
Waist circumference, cm	90.4 (13.4)	92.6 (13.5)
Physical activity (METs), h/week	44.3 (45.4)	43.4 (45.3)
Smoking status (%)		
Never	55.0	49.7
Previous	33.9	38.5
Current	10.5	11.2
Missing	0.5	0.6
Alcohol drinking intensity (%)		
Never	19.4	16.4
1–3 times/month	11.2	10.5
1–2 times/week	25.9	25.6
3–4 times/week	23.2	23.6
Daily	20.2	23.6
Missing	0.2	0.2
Processed meat intake (%)		
Never	9.3	7.1
Less than once a week	30.2	27.9
Once a week	29.0	28.2
Twice or more a week	31.3	36.6
Missing	0.3	0.1
History of cardiovascular diseases (%)		
No	70.3	67.1
Yes	29.3	32.6
Missing	0.4	0.4
History of diabetes (%)		
No	94.4	93.0
Yes	5.1	6.6
Missing	0.4	0.4
Family history of colorectal cancer (%)		
No	86.6	82.7
Yes	10.7	14.8
Missing	2.6	2.5
Colorectal cancer screening (%)		
No	68.9	73.2
Yes	29.3	25.4
Missing	1.9	1.4
Aspirin use (%)	14.0	14.2
No	84.9	84.3
Yes	13.8	13.9
Missing	1.3	1.7
Fasting status		
No	95.8	95.3
Yes	4.2	4.7
High-density cholesterol, mmol/L	1.4 (0.4)	1.4 (0.4)
Low-density cholesterol, mmol/L	3.6 (0.9)	3.6 (0.9)
Total cholesterol, mmol/L	5.7 (1.1)	5.7 (1.1)
Triglycerides, mmol/L	1.7 (0.9)	1.8 (1.0)
Apolipoprotein A, g/L	1.5 (0.3)	1.5 (0.3)
Apolipoprotein B, g/L	1.0 (0.2)	1.0 (0.2)

*ApoA* apolipoprotein A, *ApoB* apolipoprotein B, *BMI* body mass index, *HDL* high-density cholesterol, *LDL* low-density cholesterol, *MET* metabolic equivalents, *TC* total cholesterol, *TG* triglycerides.

The age-adjusted serum lipid and lipoprotein levels appeared to be similar between CRC cases and non-cases. The correlation matrix between BMI, waist circumference and lipid biomarkers showed that they were significantly correlated among both cases and non-cases (Supplemental Table 1). A high ICC (>0.60 for all biomarkers) was found in serum lipids between the initial and repeated assessments among 14,092 participants who did not develop cancer during the interval of the two measurements (Supplementary Table 2).

No statistically significant associations were found between serum lipid and lipoprotein levels and risk of CRC in the fully adjusted model (Table 2). For TG, each 1-SD increment was associated with 6% increased risk of CRC after multivariable adjustment (HR, 1.06 [95% CI, 1.02–1.10]). However, this association was attenuated to null after further adjusting for BMI and waist circumference (HR, 1.04 [95% CI, 0.99–1.08]). To assess the influence of reverse causality, we excluded the first 2 and 5 years of follow-up respectively and the results remained essentially unchanged (Supplemental Table 3). Furthermore, the results did not essentially change in the complete case analysis (Supplemental Table 4) and after additionally adjusting for other risk factors (Supplemental Table 5).

**Table 2:** Hazard ratios (95% CIs) for the associations between serum lipid levels and colorectal cancer risk in UK Biobank.

	Quintile 1	Quintile 2	Quintile 3	Quintile 4	Quintile 5	HR (95% CI) per 1-SD increase	*P* for trend
HDL							
Median (IQR)	1.00 (0.15)	1.22 (0.01)	1.39 (0.01)	1.60 (0.12)	1.94 (0.29)		
No. of cases	620	555	540	450	502		
No. of person years	745,884	754,461	749,451	751,863	751,227		
Model 1, HR (95% CI)^a^	1	0.95 (0.85–1.07)	0.97 (0.86–1.10)	0.86 (0.76–0.98)	0.97 (0.85–1.10)	0.97 (0.93–1.01)	0.19
Model 2, HR (95% CI)^b^	1	0.96 (0.85–1.09)	0.98 (0.87–1.11)	0.87 (0.76–1.00)	0.97 (0.84–1.12)	0.97 (0.93–1.02)	0.23
Model 3, HR (95% CI)^c^	1	0.98 (0.87–1.11)	1.02 (0.90–1.15)	0.92 (0.80–1.06)	1.06 (0.91–1.22)	1.00 (0.95–1.05)	0.99
LDL							
Median (IQR)	2.48 (0.45)	3.08 (0.24)	3.52 (0.22)	3.98 (0.26)	4.68 (0.59)		
No. of cases	611	505	476	523	552		
No. of person years	741,739	750,805	753,542	753,216	753,584		
Model 1, HR (95% CI)^a^	1	0.89 (0.78–1.00)	0.94 (0.84–1.07)	1.00 (0.89–1.13)	0.96 (0.85–1.09)	1.00 (0.96–1.04)	0.99
Model 2, HR (95% CI)^b^	1	0.91 (0.80–1.04)	0.97 (0.86–1.11)	1.02 (0.90–1.16)	0.96 (0.85–1.09)	1.01 (0.97–1.05)	0.63
Model 3, HR (95% CI)^c^	1	0.91 (0.80–1.03)	0.96 (0.85–1.10)	1.00 (0.88–1.14)	0.97 (0.85–1.11)	1.00 (0.96–1.05)	0.90
TG							
Median (IQR)	0.79 (0.20)	1.12 (0.16)	1.48 (0.20)	1.97 (0.31)	2.98 (1.02)		
No. of cases	421	482	534	580	650		
No. of person years	754,111	747,998	750,213	750,127	750,437		
Model 1, HR (95% CI)^a^	1	0.97 (0.85–1.11)	1.00 (0.87–1.14)	1.05 (0.92–1.19)	1.20 (1.06–1.36)	1.08 (1.04–1.12)	<0.0001
Model 2, HR (95% CI)^b^	1	0.97 (0.85–1.11)	0.99 (0.86–1.13)	1.02 (0.90–1.17)	1.15 (1.01–1.31)	1.06 (1.02–1.10)	0.002
Model 3, HR (95% CI)^c^	1	0.94 (0.82–1.08)	0.94 (0.83–1.08)	0.96 (0.84–1.10)	1.07 (0.94–1.22)	1.04 (0.99–1.08)	0.05
TC							
Median (IQR)	4.27 (0.62)	5.07 (0.31)	5.64 (0.28)	6.23 (0.33)	7.14 (0.77)		
No. of cases	618	503	480	516	550		
No. of person years	742,865	751,692	751,943	753,762	752,623		
Model 1, HR (95% CI)^a^	1	0.95 (0.84–1.08)	0.92 (0.81–1.04)	0.94 (0.83–1.06)	1.01 (0.89–1.14)	1.01 (0.97–1.05)	0.74
Model 2, HR (95% CI)^b^	1	0.97 (0.86–1.10)	0.94 (0.83–1.07)	0.96 (0.85–1.10)	1.03 (0.90–1.17)	1.02 (0.97–1.06)	0.48
Model 3, HR (95% CI)^c^	1	0.97 (0.86–1.10)	0.94 (0.83–1.08)	0.96 (0.84–1.10)	1.02 (0.89–1.16)	1.01 (0.97–1.06)	0.59
ApoA							
Median (IQR)	1.22 (0.12)	1.38 (0.07)	1.51 (0.06)	1.65 (0.08)	1.90 (0.23)		
No. of cases	579	547	526	510	505		
No. of person years	746,223	754,022	749,910	754,453	748,278		
Model 1, HR (95% CI)^a^	1	0.95 (0.84–1.07)	0.94 (0.83–1.06)	0.95 (0.84–1.08)	0.95 (0.83–1.08)	0.99 (0.95–1.03)	0.64
Model 2, HR (95% CI)^b^	1	0.96 (0.85–1.08)	0.94 (0.83–1.07)	0.95 (0.83–1.08)	0.93 (0.81–1.07)	0.98 (0.94–1.03)	0.46
Model 3, HR (95% CI)^c^	1	0.97 (0.86–1.09)	0.96 (0.85–1.09)	0.98 (0.86–1.12)	0.99 (0.85–1.14)	1.00 (0.96–1.05)	0.93
ApoB							
Median (IQR)	0.74 (0.12)	0.90 (0.06)	1.02 (0.06)	1.14 (0.07)	1.34 (0.18)		
No. of cases	585	484	496	534	568		
No. of person years	743,244	754,942	747,569	755,792	751,338		
Model 1, HR (95% CI)^a^	1	0.85 (0.75–0.96)	0.89 (0.79–1.01)	0.93 (0.82–1.05)	0.99 (0.88–1.12)	1.02 (0.98–1.06)	0.24
Model 2, HR (95% CI)^b^	1	0.86 (0.75–0.97)	0.91 (0.80–1.03)	0.95 (0.83–1.07)	1.00 (0.89–1.14)	1.03 (0.99–1.07)	0.16
Model 3, HR (95% CI)^c^	1	0.85 (0.75–0.97)	0.90 (0.79–1.02)	0.93 (0.82–1.05)	0.97 (0.86–1.10)	1.02 (0.98–1.06)	0.38

The units of HDL, LDL, TC and TG are mmol/L; the units of ApoA and ApoB are g/L.

*ApoA* apolipoprotein A, *ApoB* apolipoprotein B, *CI* confidence interval, *HDL* high-density cholesterol, *HR* hazard ratio, *IQR* interquartile range, *LDL* low-density cholesterol, *TC* total cholesterol, *TG* triglycerides.

^a^Cox proportional hazards regression model with age as the time scale was adjusted for age, sex and race (Model 1).

^b^Model 2 was further adjusted for Townsend index, height, smoking status, alcohol drinking, physical activity, processed meat intake, fasting status, family history of colorectal cancer, aspirin use, history of cardiovascular diseases, history of diabetes and colorectal cancer screening.

^c^Model 3 was further adjusted for BMI and waist circumference.

In the CRC subsite analysis using the fully adjusted model (Table 3), we found a positive association of TG with cancers in the caecum and transverse colon, with the HR per SD of 1.12 [95% CI, 1.00–1.25] and 1.28 [95% CI, 1.08–1.53], respectively; an inverse association was found between ApoA and cancer in the hepatic flexure (HR, 0.73 [95% CI, 0.56–0.96]). In addition, LDL and TC showed a non-significant positive association with transverse colon cancer (HR, 1.15 [95% CI, 0.95–1.40]; HR, 1.19 [95% CI, 0.98–1.45], respectively), while HDL showed an inverse association with risk of cancer in the hepatic flexure (HR, 0.77 [95% CI, 0.59–1.03]). None of the lipids and lipoproteins showed a statistically significant heterogeneity in the association with subsite-specific CRC (*P* for heterogeneity > 0.005).

**Table 3:** Hazard ratios (95% CIs) of colorectal cancer risk per 1-SD increment in serum lipid levels according to cancer subsites and time of onset (Adjusted for age, sex, race, Townsend index, height, BMI, waist circumference, smoking status, alcohol drinking, physical activity, processed meat intake, family history of colorectal cancer, aspirin use, history of cardiovascular diseases, history of diabetes and colorectal cancer screening. Ninety-three patients with overlapping or unspecified subsites were not considered in the analysis)

Subsite	HDL	LDL	TC	TG	ApoA	ApoB
By CRC subsites						
Colon (*n* = 1 757)	0.97 (0.91–1.03)	1.00 (0.95–1.05)	1.00 (0.95–1.06)	1.06 (1.01–1.11)	0.97 (0.92–1.03)	1.01 (0.96–1.07)
Caecum (*n* = 342)	0.99 (0.86–1.13)	1.02 (0.91–1.15)	1.04 (0.92–1.16)	1.12 (1.00–1.25)	1.02 (0.90–1.16)	1.02 (0.91–1.14)
Ascend (*n* = 231)	0.98 (0.83–1.16)	1.02 (0.89–1.17)	1.04 (0.90–1.19)	1.10 (0.97–1.25)	1.01 (0.86–1.18)	1.05 (0.91–1.20)
Hepatic flexure (*n* = 93)	0.77 (0.58–1.03)	0.94 (0.75–1.18)	0.91 (0.72–1.14)	1.03 (0.84–1.28)	0.73 (0.56–0.96)	0.97 (0.78–1.21)
Transverse (*n* = 119)	0.91 (0.72–1.15)	1.15 (0.95–1.40)	1.19 (0.98–1.45)	1.28 (1.08–1.52)	0.91 (0.72–1.14)	1.19 (0.98–1.44)
Splenic flexure (*n* = 63)	0.80 (0.57–1.12)	0.85 (0.64–1.13)	0.81 (0.61–1.07)	0.86 (0.64–1.15)	0.79 (0.57–1.09)	0.88 (0.67–1.16)
Descend (*n* = 94)	0.96 (0.74–1.26)	0.89 (0.71–1.12)	0.86 (0.68–1.09)	0.89 (0.70–1.13)	0.83 (0.64–1.08)	0.98 (0.78–1.22)
Sigmoid (*n* = 676)	1.04 (0.94–1.14)	1.01 (0.93–1.10)	1.02 (0.94–1.11)	1.05 (0.97–1.13)	1.04 (0.94–1.14)	1.02 (0.94–1.11)
Rectum (*n* = 910)	1.06 (0.98–1.15)	1.01 (0.94–1.09)	1.03 (0.96–1.11)	1.01 (0.94–1.08)	1.06 (0.98–1.15)	1.03 (0.96–1.10)
*P* for heterogeneity	0.34	0.71	0.33	0.06	0.08	0.80
By age of CRC onset						
Early-onset (<50 years) (*n* = 92)	1.06 (0.81–1.39)	1.03 (0.81–1.31)	1.05 (0.83–1.34)	0.93 (0.73–1.18)	1.07 (0.83–1.38)	0.99 (0.78–1.25)
Middle-onset (50–59 years) (*n* = 508)	1.01 (0.90–1.13)	1.04 (0.94–1.14)	1.04 (0.95–1.15)	1.08 (0.99–1.18)	1.01 (0.91–1.13)	1.07 (0.97–1.17)
Late-onset (≥60) (*n* = 2067)	1.00 (0.95–1.05)	1.00 (0.96–1.05)	1.01 (0.97–1.06)	1.05 (1.01–1.09)	1.00 (0.95–1.05)	1.02 (0.98–1.07)
*P* for heterogeneity	0.89	0.84	0.83	0.52	0.85	0.69

The units of HDL, LDL, TC and TG are mmol/L; the units of ApoA and ApoB are g/L.

*ApoA* apolipoprotein A, *ApoB* apolipoprotein B, *CRC* colorectal cancer, *HDL* high-density cholesterol, *LDL* low-density cholesterol, *TC* total cholesterol, *TG* triglycerides.

In the analysis according to age of CRC onset (Table 3), no substantial difference was found in the associations of lipids with risk of early-, middle-, and late-onset CRC (*P* for heterogeneity > 0.005). For the stratified analysis according to CRC risk factors (Supplemental Table 6), no statistically significant interaction was found using the stringent *P* value < 0.005 as the cut-off.

## Discussion

In this large prospective study of 2667 cases of incident CRC, after adjusting for potential confounding factors including BMI and waist circumference, we did not find any statistically significant association of pre-diagnostic concentrations of serum lipids and lipoproteins (HDL, LDL, TC, TG, ApoA, ApoB) with CRC risk. However, a suggested association was found for TG with higher risk of caecum and transverse colon cancer risk and ApoA with lower risk of cancer in the hepatic flexure.

The relation between lipid biomarkers and CRC risk has been previously examined in other population-based studies but the results were highly inconsistent. A nested case−control study with 1238 first-incident CRC cases and 1:1 matched controls based on European Prospective Investigation into Cancer and Nutrition (EPIC) showed that high concentrations of serum HDL and ApoA were associated with a decreased risk of colon cancer, while no associations were observed with the risk of rectal cancer.^42^ Such inconsistency with our study may be contributed by its short follow-up period (mean: 3.8 years), higher concentrations of baseline lipids/lipoproteins in the study base and residual confounding due to incomplete adjustment. In a prospective cohort study among 26,408 participants in Sweden, risk of CRC was positively associated with the level of ApoB, but not ApoA, HDL or LDL.^12^ The studies with smaller sample sizes are subject to chance findings. Another large prospective Swedish study observed a significant positive association between TG and colon cancer risk.^43^ Of note, none of the prior studies adjusted for waist circumference and are thus prone to residual confounding by abdominal obesity. In addition, there are emerging body of evidence on lipids and CRC using Mendelian randomisation approach. A two-sample Mendelian randomisation study examining the relationship between 39 potentially modifiable risk factors and CRC found none of the HDL, LDL, TC, and TG were related to CRC risk after correction for multiple testing, but suggested a non-significant relationship between LDL (OR 1.14 [95% CI 1.04–1.25]; *P* = 0.0056) and increased CRC risk.^44^ Another study using genetic risk score derived from 119 genetic variants controls did not find any association of HDL, LDL, TC and TG with the risk of CRC, indicating that lifetime dyslipidaemia most probably is unrelated to the colorectal neoplasms.^45^

Extensive studies including systematic review and meta-analysis have reported evidence of increased risk of CRC associated with general and abdominal obesity. However, the exact biologic mechanism underlying the relationship has not been fully elucidated.^16,46–49^ In contrast with general obesity, body fat mass distribution—particularly abdominal obesity—appears to be more predictive of CRC risk, which is linked to insulin resistance and hyperinsulinemia.^48,50,51^ Experimental and epidemiologic studies have demonstrated that adiposity promotes the production of a variety of hormones and pro-inflammatory cytokines, including IL-6, TNF-α and C-peptide.^52–55^ Accumulating evidence suggests that insulin resistance represents the most plausible link between obesity and dyslipidaemia featured by lower HDL and higher TG, which may lead to adiposopathy, increased lipolysis and release of FFAs into the circulation.^56–58^ Given that impaired production of adipokines and chronic low-grade inflammation in adipose tissue forming the base for insulin resistance is the main driving force in the development of metabolic dyslipidaemia, the null associations between serum lipid profiles and CRC after adjusting for waist circumference and BMI are not unexpected.^56,57^

Recent investigations have highlighted the subsite heterogeneity of CRC in terms of the embryologic origins, associated microbial milieu, immune environment and tumour characteristics such as mutational signatures and molecular features.^59–61^ Interestingly, in the present study, hepatic flexure and transverse colon cancer were observed to be associated with serum lipids and lipoproteins. Although no study has yet assessed lipids with refined subsite-specific risk of CRC, the empirical dietary index for hyperinsulinemia was reported to be more strongly associated with increased risk of transverse (including hepatic flexure) and descending colon cancer, possibly through the IGF1-PI3K/AKT pathway.^21^ Increasing evidence shows that obesity-related abnormalities may influence cancer risk through alterations of the microbiome that differ substantially across the colorectum.^62^ Microbiota can metabolise nutrients (including lipids) for the production of inflammatory and/or carcinogenic metabolites, thereby increasing proliferation and suppressing apoptosis through effects on immunity, gene expression and epigenetic modulation.^63^ Therefore, our subsite findings may be explained by the different distribution of the gut microbiota across the colorectum. Indeed, a recent study showed that statin can restore the microbial alterations induced by obesity and exert a beneficial effect on host metabolism.^64^ However, given the limited data, further studies are needed to better understand the potential role of lipids in subsite-specific risk of CRC.

The main strengths of this study include its prospective design, large sample size, relatively long follow-up period and comprehensive assessment of covariates including anthropometric measures, lifestyle, medical history and medication use. Moreover, all lipid biomarkers were measured using the standardised and validated blood biochemistry methods with strict quality controls in a single central laboratory, thereby minimising any measurement errors.^34^ In the secondary analyses, multiple testing due to multiple subgroup comparisons could potentially inflate type I error (i.e. the probability of rejecting at least one null hypothesis given that all nulls are in fact true). To address the issue, we adopted a more stringent type I error threshold (0.005) for the heterogeneity test and interaction test. Some limitations of the study should also be considered. First, a single baseline measurement of lipids and lipoproteins was used for the main analysis and thus susceptible to short-term variation. However, the high ICC in a subsample with repeated assessments indicated that time-dependent variation in lipids was unlikely to have a substantial impact on our results. Secondly, given the long process of CRC development, it is possible that early carcinogenesis may induce metabolic changes in the lipid profiles that may have distorted the association between lipids and CRC risk. However, our sensitivity analysis of excluding cases diagnosed within the first 2 and 5 years after blood draw did not reveal any substantial changes in the results. In addition, due to the age structure of UK Biobank, the majority of participants entered the cohort after 50 years old. Therefore, the statistical power to detect associations with early-onset CRC is limited. While UK Biobank participants are not representative of the general population, valid assessment of exposure−disease relationships are nonetheless widely generalisable and do not require participants to be representative of the population at large. Finally, we only assessed circulating lipids. It may be the local biochemical changes in the gut environment that are more important to CRC development.

In conclusion, in this large prospective cohort study, we observed no associations between serum lipid profiles and CRC risk after adjusting for obesity indicators. The suggestive associations of triglyceride and ApoA with hepatic flexure and transverse colon cancer require further confirmation.

## Supplementary information

Supplemental table 1-6

## Data Availability

Approval for the study and permission to access the data was granted by the UK Biobank Resource under Application Number 46466 that is open access. Bona fide researchers can access the UK Biobank dataset by registering and applying at http://ukbiobank.ac.uk/register-apply/.
