# Supplementary material for: Serum lipid profiles and risk of colorectal cancer: a prospective cohort study in the UK Biobank
Source: Br J Cancer. 2020 Nov 3;124(3):663–70. doi: 10.1038/s41416-020-01143-6 (PMC7851156; doi:10.1038/s41416-020-01143-6)
Supplement: Supplementary file 1 — Supplemental table 1-6 [file 41416_2020_1143_MOESM1_ESM.docx]

**Supplemental table 1 The correlation matrix between BMI, waist circumference and lipid biomarkers**

| CRC cases | Pearson correlation coefficient (P value) | |
| --- | --- | --- |
|  | BMI | Waist Circumference |
| HDL | -0.37(0.0001) | -0. 47(<0.0001) |
| LDL | -0.06(0.002) | -0.14(<0.0001) |
| TC | -0.13(<0.0001) | -0.22(<0.0001) |
| TG | 0.30(<0.0001) | 0.34(<0.0001) |
| ApoA | -0.29(<0.0001) | -0.39(<0.0001) |
| ApoB | 0.01(0.58) | -0.05(0.02) |
| CRC non-cases |  |  |
|  | BMI | Waist Circumference |
| HDL | -0.35(<0.0001) | -0.48(<0.0001) |
| LDL | 0.02(<0.0001) | -0.06(0.002) |
| TC | -0.05(<0.0001) | -0.10(<0.0001) |
| TG | 0.29(<0.0001) | 0.37(<0.0001) |
| ApoA | -0.27(<0.0001) | -0.38(<0.0001) |
| ApoB | 0.08(<0.0001) | 0.08(<0.0001) |

Abbreviations: ApoA, apolipoprotein A; ApoB, apolipoprotein B; CI, confidence interval; CRC, colorectal cancer; HDL, high-density cholesterol; HR, hazard ratio; IQR, interquartile range; LDL, low-density cholesterol; TC, total cholesterol; TG, triglycerides.

**Supplemental table 2 Intraclass correlation coefficient between two repeated measurements of lipid biomarkers**

|  | HDL | LDL | TC | TG | ApoA | ApoB |
| --- | --- | --- | --- | --- | --- | --- |
| No. of participants | 12 070 | 13 061 | 13 100 | 13 093 | 12 036 | 13 054 |
| ICC (95% CI) | 0.84 (0.84, 0.85) | 0.65 (0.64, 0.66) | 0.66 (0.65, 0.67) | 0.61 (0.60, 0.62) | 0.77 (0.76, 0.78) | 0.66 (0.65, 0.67) |

Abbreviations: ApoA, apolipoprotein A; ApoB, apolipoprotein B; CI, confidence interval; HDL, high-density cholesterol; ICC, intraclass correlation coefficient; LDL, low-density cholesterol; TC, total cholesterol; TG, triglycerides.

**Supplemental table 3 Hazard ratios (95% CIs) for the associations between serum lipid levels and colorectal cancer risk in UK Biobank after exclusions of cases diagnosed within 2 years (N=379 440) and 5 years (N=378 301) after blood draw**

|  | HDL | LDL | TC | TG | ApoA | ApoB |
| --- | --- | --- | --- | --- | --- | --- |
| Exclude cases within 2 years |  |  |  |  |  |  |
| Model 1, HR (95% CI)^a^ | 0.96 (0.91-1.01) | 1.00 (0.96-1.04) | 1.01 (0.96-1.05) | 1.09 (1.04-1.13) | 0.98 (0.93-1.03) | 1.02 (0.97-1.06) |
| Model 2, HR (95% CI)^b^ | 0.96 (0.91-1.01) | 1.01 (0.96-1.06) | 1.01 (0.97-1.06) | 1.07 (1.03-1.12) | 0.97 (0.92-1.02) | 1.02 (0.98-1.07) |
| Model 3, HR (95% CI)^c^ | 1.00 (0.94-1.05) | 1.00 (0.95-1.05) | 1.01 (0.96-1.06) | 1.04 (1.00-1.09) | 1.00 (0.95-1.05) | 1.01 (0.96-1.06) |
| Exclude cases within 5 years |  |  |  |  |  |  |
| Model 1, HR (95% CI)^a^ | 0.95 (0.88-1.02) | 0.99 (0.93-1.06) | 1.00 (0.93-1.07) | 1.09 (1.02-1.16) | 0.98 (0.91-1.05) | 1.00 (0.94-1.07) |
| Model 2, HR (95% CI)^b^ | 0.94 (0.87-1.02) | 1.00 (0.93-1.07) | 1.00 (0.93-1.08) | 1.07 (1.00-1.14) | 0.96 (0.89-1.04) | 1.01 (0.94-1.08) |
| Model 3, HR (95% CI)^c^ | 0.97 (0.89-1.05) | 0.99 (0.92-1.06) | 1.00 (0.93-1.07) | 1.05 (0.98-1.12) | 0.98 (0.91-1.06) | 0.99 (0.93-1.07) |

Abbreviations: ApoA, apolipoprotein A; ApoB, apolipoprotein B; CI, confidence interval; HDL, high-density cholesterol; HR, hazard ratio; IQR, interquartile range; LDL, low-density cholesterol; TC, total cholesterol; TG, triglycerides.

The units of HDL, LDL, TC and TG are mmol/l; the units of ApoA and ApoB are g/l.

^a^ Cox proportional hazards regression model with age as the time scale was adjusted for age, sex and race (Model 1).

^b^ Model 2 was further adjusted for Townsend index, height, smoking status, alcohol drinking, physical activity, processed meat intake, fasting status, family history of colorectal cancer, aspirin use, history of cardiovascular diseases, history of diabetes and colorectal cancer screening.

^c^ Model 3 was further adjusted for BMI and waist circumference.

**Supplemental table 4 Hazard ratios (95% CIs) for the associations between serum lipid levels and colorectal cancer risk in UK Biobank after exclusions of participants with missing covariates (N=291 218)**

|  | HDL | LDL | TC | TG | ApoA | ApoB |
| --- | --- | --- | --- | --- | --- | --- |
| Model 1, HR (95% CI)^a^ | 0.97 (0.92-1.02) | 1.01 (0.97-1.06) | 1.02 (0.98-1.07) | 1.09 (1.04-1.13) | 0.99 (0.94-1.04) | 1.04 (0.99-1.09) |
| Model 2, HR (95% CI)^b^ | 1.00 (0.94-1.05) | 1.02 (0.97-1.07) | 1.03 (0.98-1.08) | 1.05 (1.00-1.10) | 1.00 (0.95-1.06) | 1.04 (0.99-1.09) |
| Model 3, HR (95% CI)^c^ | 1.00 (0.94-1.05) | 1.02 (0.97-1.07) | 1.03 (0.98-1.08) | 1.05 (1.00-1.10) | 1.00 (0.95-1.06) | 1.04 (0.99-1.09) |

Abbreviations: ApoA, apolipoprotein A; ApoB, apolipoprotein B; CI, confidence interval; HDL, high-density cholesterol; HR, hazard ratio; IQR, interquartile range; LDL, low-density cholesterol; TC, total cholesterol; TG, triglycerides.

The units of HDL, LDL, TC and TG are mmol/l; the units of ApoA and ApoB are g/l.

^a^ Cox proportional hazards regression model with age as the time scale was adjusted for age, sex and race (Model 1).

^b^ Model 2 was further adjusted for Townsend index, height, smoking status, alcohol drinking, physical activity, processed meat intake, fasting status, family history of colorectal cancer, aspirin use, history of cardiovascular diseases, history of diabetes, fasting status and colorectal cancer screening.

^c^ Model 3 was further adjusted for BMI and waist circumference.

**Supplemental table 5 Hazard ratios (95% CIs) of colorectal cancer risk per 1-SD increment in serum lipid levels further adjusting for HRT use and dietary factors ^a^**

|  | Quintile 1 | Quintile 2 | Quintile 3 | Quintile 4 | Quintile 5 | HR (95% CI) per 1-SD increase | P for trend |
| --- | --- | --- | --- | --- | --- | --- | --- |
| **HDL** |  |  |  |  |  |  |  |
| Median (IQR) | 1.00 (0.15) | 1.22 (0.01) | 1.39 (0.01) | 1.60 (0.12) | 1.94 (0.29) |  |  |
| No. of cases | 620 | 555 | 540 | 450 | 502 |  |  |
| No. of person years | 745 884 | 754 461 | 749 451 | 751 863 | 751 227 |  |  |
| Model 4, HR (95% CI) | 1 | 0.99 (0.87-1.11) | 1.02 (0.90-1.16) | 0.93 (0.81-1.07) | 1.06 (0.91-1.23) | 1.00 (0.95-1.05) | 0.95 |
| **LDL** |  |  |  |  |  |  |  |
| Median (IQR) | 2.48 (0.45) | 3.08 (0.24) | 3.52 (0.22) | 3.98 (0.26) | 4.68 (0.59) |  |  |
| No. of cases | 611 | 505 | 476 | 523 | 552 |  |  |
| No. of person years | 741 739 | 750 805 | 753 542 | 753 216 | 753 584 |  |  |
| Model 4, HR (95% CI) | 1 | 0.96 (0.85-1.09) | 0.91 (0.79-1.03) | 0.96 (0.84-1.09) | 1.00 (0.88-1.14) | 1.00 (0.96-1.04) | 0.94 |
| **TG** |  |  |  |  |  |  |  |
| Median (IQR) | 0.79(0.20) | 1.12(0.16) | 1.48(0.20) | 1.97(0.31) | 2.98(1.02) |  |  |
| No. of cases | 421 | 482 | 534 | 580 | 650 |  |  |
| No. of person years | 754 111 | 747 998 | 750 213 | 750 127 | 750 437 |  |  |
| Model 4, HR (95% CI) | 1 | 0.94 (0.82-1.08) | 0.94 (0.82-1.08) | 0.96 (0.84-1.10) | 1.07 (0.93-1.22) | 1.04 (1.00-1.08) | 0.06 |
| **TC** |  |  |  |  |  |  |  |
| Median (IQR) | 4.27 (0.62) | 5.07 (0.31) | 5.64 (0.28) | 6.23 (0.33) | 7.14 (0.77) |  |  |
| No. of cases | 618 | 503 | 480 | 516 | 550 |  |  |
| No. of person years | 742 865 | 751 692 | 751 943 | 753 762 | 752 623 |  |  |
| Model 4, HR (95% CI) | 1 | 0.97 (0.86-1.10) | 0.94 (0.82-1.07) | 0.96 (0.84-1.10) | 1.01 (0.89-1.16) | 1.01 (0.97-1.05) | 0.62 |
| **ApoA** |  |  |  |  |  |  |  |
| Median (IQR) | 1.22 (0.12) | 1.38 (0.07) | 1.51 (0.06) | 1.65 (0.08) | 1.90 (0.23) |  |  |
| No. of cases | 579 | 547 | 526 | 510 | 505 |  |  |
| No. of person years | 746 223 | 754 022 | 749 910 | 754 453 | 748 278 |  |  |
| Model 4, HR (95% CI) | 1 | 0.97 (0.86-1.10) | 0.97 (0.85-1.10) | 0.99 (0.86-1.13) | 0.99 (0.86-1.14) | 1.00 (0.96-1.05) | 0.89 |
| **ApoB** |  |  |  |  |  |  |  |
| Median (IQR) | 0.74 (0.12) | 0.90 (0.06) | 1.02 (0.06) | 1.14 (0.07) | 1.34 (0.18) |  |  |
| No. of cases | 585 | 484 | 496 | 534 | 568 |  |  |
| No. of person years | 743 244 | 754 942 | 747 569 | 755 792 | 751 338 |  |  |
| Model 4, HR (95% CI) | 1 | 0.85 (0.75-0.96) | 0.89 (0.79-1.02) | 0.92 (0.81-1.05) | 0.97 (0.85-1.10) | 1.02 (0.98-1.06) | 0.41 |

Abbreviations: ApoA, apolipoprotein A; ApoB, apolipoprotein B; CI, confidence interval; HDL, high-density cholesterol; HR, hazard ratio; HRT, hormone replacement therapy; IQR, interquartile range; LDL, low-density cholesterol; TC, total cholesterol; TG, triglycerides.

The units of HDL, LDL, TC and TG are mmol/l; the units of ApoA and ApoB are g/l.

^a^ Model 4 was adjusted for age, sex, race, Townsend index, height, smoking status, alcohol drinking, physical activity, processed meat intake, fasting status, family history of colorectal cancer, aspirin use, history of cardiovascular diseases, history of diabetes, colorectal cancer screening, BMI, waist circumference, HRT use (women only), and intake of red meat (beef, lamb and pork) intake, fruit and vegetable.

**Supplemental table 6 Hazard ratios (95% CIs) of colorectal cancer risk per 1-SD increment in serum lipid levels according to risk factors**

| Stratified variable | No. of cases | HDL | LDL | TC | TG | ApoA | ApoB |
| --- | --- | --- | --- | --- | --- | --- | --- |
| **Age** |  |  |  |  |  |  |  |
| <50 | 190 | 1.09 (0.91-1.30) | 1.09 (0.92-1.28) | 1.11 (0.94-1.31) | 1.00 (0.85-1.17) | 1.07 (0.90-1.28) | 1.07 (0.91-1.25) |
| 50-60 | 731 | 1.00 (0.91-1.09) | 1.01 (0.93-1.09) | 1.02 (0.94-1.10) | 1.07 (1.00-1.15) | 1.01 (0.92-1.10) | 1.03 (0.96-1.12) |
| ≥60 | 1 746 | 1.00 (0.94-1.06) | 1.01 (0.96-1.07) | 1.02 (0.97-1.08) | 1.05 (0.99-1.10) | 1.00 (0.94-1.06) | 1.03 (0.98-1.08) |
| P for interaction |  | 0.006 | 0.33 | 0.17 | 0.17 | 0.01 | 0.40 |
| **Sex** |  |  |  |  |  |  |  |
| Female | 1 112 | 0.98 (0.91-1.05) | 1.04 (0.97-1.10) | 1.04 (0.98-1.11) | 1.08 (1.01-1.16) | 0.99 (0.93-1.06) | 1.05 (0.98-1.11) |
| Male | 1 555 | 1.02 (0.95-1.09) | 0.99 (0.94-1.05) | 1.00 (0.95-1.06) | 1.03 (0.98-1.08) | 1.01 (0.95-1.08) | 1.01 (0.96-1.07) |
| P for interaction |  | 0.54 | 0.36 | 0.49 | 0.94 | 0.49 | 0.62 |
| **BMI category** |  |  |  |  |  |  |  |
| <25 kg/m^2^ | 711 | 1.02 (0.94-1.11) | 0.96 (0.88-1.04) | 0.97 (0.89-1.06) | 0.90 (0.81-1.01) | 1.02 (0.94-1.11) | 0.96 (0.88-1.05) |
| 25-30 kg/m^2^ | 1 231 | 1.03 (0.96-1.11) | 1.01 (0.95-1.07) | 1.03 (0.96-1.09) | 1.04 (0.98-1.10) | 1.01 (0.94-1.09) | 1.02 (0.96-1.08) |
| ≥30 kg/m^2^ | 725 | 0.93 (0.84-1.04) | 1.03 (0.95-1.12) | 1.02 (0.94-1.11) | 1.09 (1.02-1.17) | 0.98 (0.89-1.08) | 1.06 (0.98-1.15) |
| P for interaction |  | 0.35 | 0.78 | 0.70 | 0.01 | 0.81 | 0.48 |
| **WC sex-specific tertiles** |  |  |  |  |  |  |  |
| Q1 | 660 | 0.96 (0.88-1.06) | 1.01 (0.92-1.10) | 1.00 (0.92-1.10) | 1.00 (0.90-1.11) | 0.99 (0.90-1.08) | 1.03 (0.94-1.12) |
| Q2 | 922 | 1.09 (1.01-1.19) | 1.06 (0.99-1.14) | 1.09 (1.01-1.17) | 1.01 (0.94-1.08) | 1.07 (0.99-1.16) | 1.06 (0.99-1.14) |
| Q3 | 1 085 | 0.95 (0.87-1.04) | 0.95 (0.89-1.01) | 0.95 (0.89-1.02) | 1.07 (1.01-1.13) | 0.96 (0.89-1.04) | 0.98 (0.92-1.04) |
| P for interaction |  | 0.16 | 0.04 | 0.03 | 0.37 | 0.31 | 0.15 |
| **Smoking status** |  |  |  |  |  |  |  |
| Never | 1 629 | 0.95 (0.89-1.01) | 1.00 (0.95-1.06) | 1.08 (1.03-1.14) | 1.01 (0.95-1.06) | 0.97 (0.91-1.03) | 1.03 (0.97-1.08) |
| Former | 318 | 1.03 (0.90-1.18) | 0.97 (0.86-1.10) | 0.95 (0.83-1.07) | 0.97 (0.85-1.09) | 0.99 (0.87-1.13) | 0.98 (0.87-1.11) |
| Current | 704 | 1.10 (1.00-1.21) | 1.01 (0.93-1.10) | 1.00 (0.93-1.08) | 1.04 (0.96-1.13) | 1.08 (0.99-1.19) | 1.02 (0.94-1.10) |
| P for interaction |  | 0.39 | 0.66 | 0.68 | 0.09 | 0.62 | 0.43 |
| **Aspirin use** |  |  |  |  |  |  |  |
| No | 2 124 | 0.99 (0.93-1.04) | 1.01 (0.97-1.06) | 1.02 (0.97-1.07) | 1.04 (1.00-1.09) | 0.99 (0.94-1.05) | 1.03 (0.98-1.08) |
| Yes | 502 | 1.04 (0.93-1.17) | 0.96 (0.87-1.06) | 0.99 (0.89-1.09) | 1.05 (0.96-1.15) | 1.03 (0.92-1.15) | 0.98 (0.88-1.08) |
| P for interaction |  | 0.92 | 0.33 | 0.59 | 0.94 | 0.96 | 0.38 |
| **Alcohol drinking** |  |  |  |  |  |  |  |
| Never | 450 | 0.90 (0.79-1.03) | 0.98 (0.89-1.09) | 0.98 (0.89-1.09) | 1.04 (0.94-1.14) | 0.94 (0.84-1.07) | 0.99 (0.89-1.09) |
| 1-3 times/month | 249 | 0.99 (0.84-1.18) | 1.02 (0.89-1.16) | 1.03 (0.90-1.19) | 1.18 (1.04-1.34) | 1.09 (0.92-1.27) | 1.04 (0.91-1.19) |
| 1-2 times/week | 660 | 0.94 (0.84-1.04) | 1.00 (0.92-1.08) | 1.00 (0.92-1.09) | 1.06 (0.98-1.15) | 0.90 (0.82-1.00) | 1.02 (0.94-1.11) |
| 3-4 times/week | 621 | 1.00 (0.90-1.11) | 1.01 (0.92-1.10) | 1.01 (0.93-1.11) | 1.02 (0.93-1.11) | 0.99 (0.89-1.09) | 1.02 (0.93-1.11) |
| Daily | 680 | 1.10 (1.01-1.20) | 1.00 (0.92-1.09) | 1.02 (0.94-1.11) | 0.99 (0.91-1.07) | 1.09 (1.01-1.19) | 1.02 (0.94-1.11) |
| P for interaction |  | 0.23 | 0.99 | 0.97 | 0.54 | 0.04 | 0.99 |
| **Physical activity** |  |  |  |  |  |  |  |
| Q1 | 547 | 1.14 (1.02-1.27) | 1.04 (0.94-1.13) | 1.06 (0.97-1.16) | 0.98 (0.90-1.07) | 1.12 (1.01-1.24) | 1.05 (0.96-1.15) |
| Q2 | 520 | 0.98 (0.87-1.10) | 1.03 (0.94-1.14) | 1.04 (0.94-1.14) | 1.05 (0.96-1.15) | 0.98 (0.88-1.09) | 1.06 (0.96-1.16) |
| Q3 | 335 | 1.10 (0.96-1.26) | 1.07 (0.95-1.21) | 1.11 (0.98-1.24) | 1.03 (0.92-1.16) | 1.10 (0.97-1.26) | 1.05 (0.94-1.18) |
| Q4 | 733 | 0.97 (0.89-1.07) | 0.92 (0.85-1.00) | 0.93 (0.85-1.00) | 1.04 (0.97-1.13) | 0.97 (0.89-1.06) | 0.95 (0.88-1.03) |
| Q5 | 532 | 0.88 (0.79-0.98) | 1.01 (0.92-1.11) | 1.01 (0.92-1.11) | 1.10 (1.01-1.20) | 0.90 (0.81-1.00) | 1.03 (0.94-1.13) |
| P for interaction |  | 0.002 | 0.24 | 0.13 | 0.18 | 0.009 | 0.33 |
| **History of CVD** |  |  |  |  |  |  |  |
| No | 1611 | 0.99 (0.93-1.06) | 1.00 (0.95-1.06) | 1.01 (0.96-1.07) | 1.04 (0.98-1.09) | 0.99 (0.94-1.06) | 1.02 (0.96-1.07) |
| Yes | 1045 | 1.02 (0.94-1.10) | 1.01 (0.94-1.08) | 1.02 (0.95-1.09) | 1.05 (0.99-1.11) | 1.02 (0.95-1.10) | 1.03 (0.96-1.10) |
| P for interaction |  | 0.54 | 0.37 | 0.49 | 0.94 | 0.49 | 0.62 |

Abbreviations: ApoA, apolipoprotein A; ApoB, apolipoprotein B; BMI, body mass index; HDL, high-density cholesterol; LDL, low-density cholesterol; Q3, tertiles; Q5, quintiles; TG, triglycerides; TC, total cholesterol; WC, waist circumference.

The units of HDL, LDL, TC and TG are mmol/l; the units of ApoA and ApoB are g/l.
